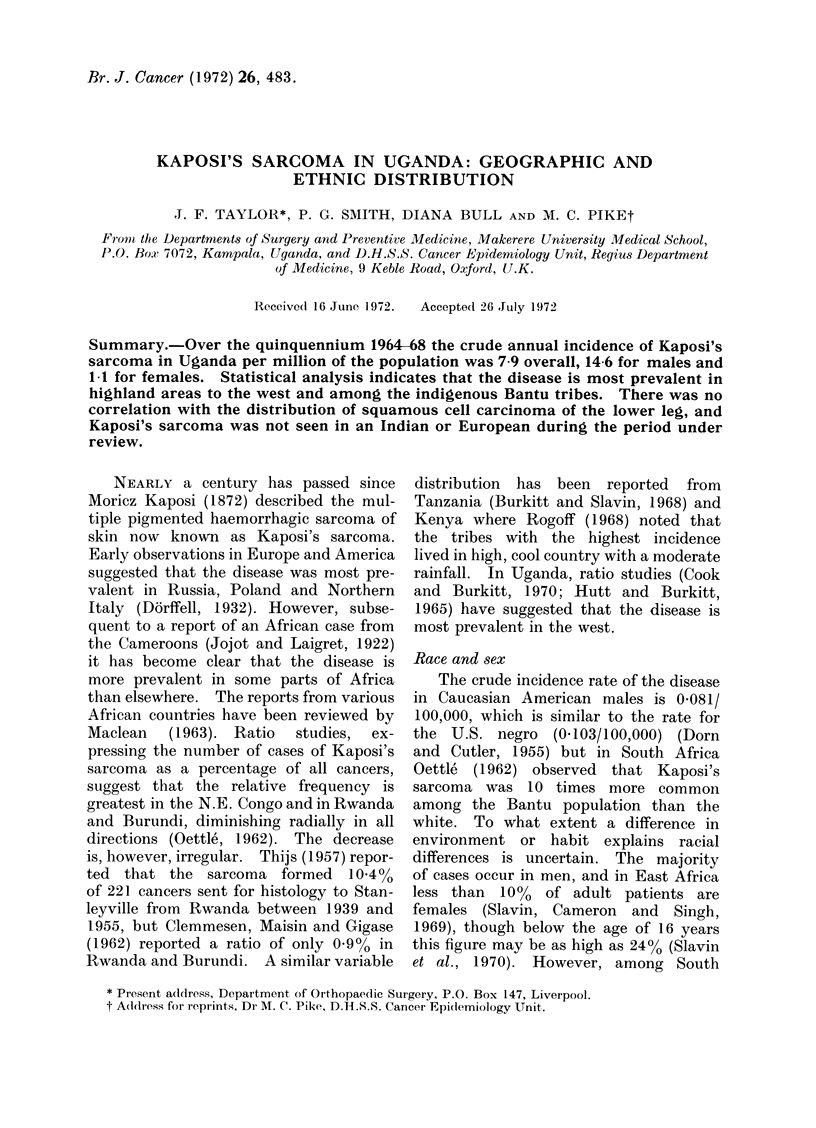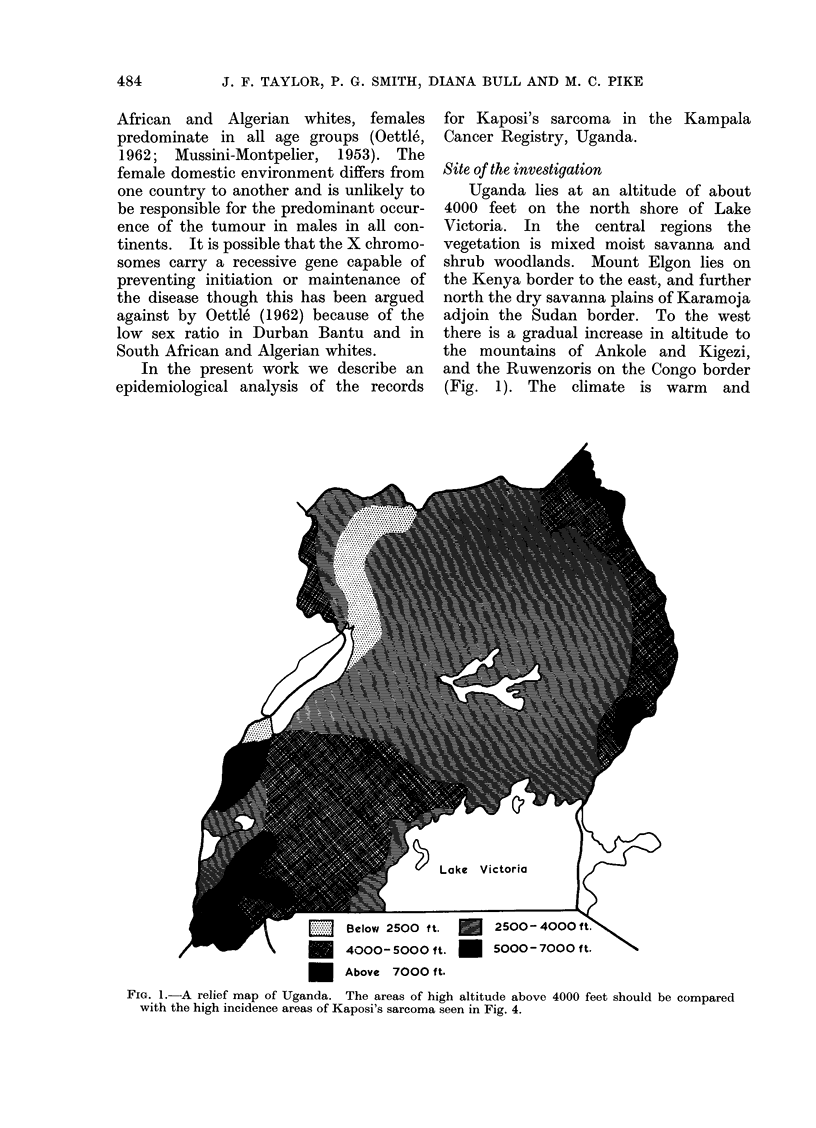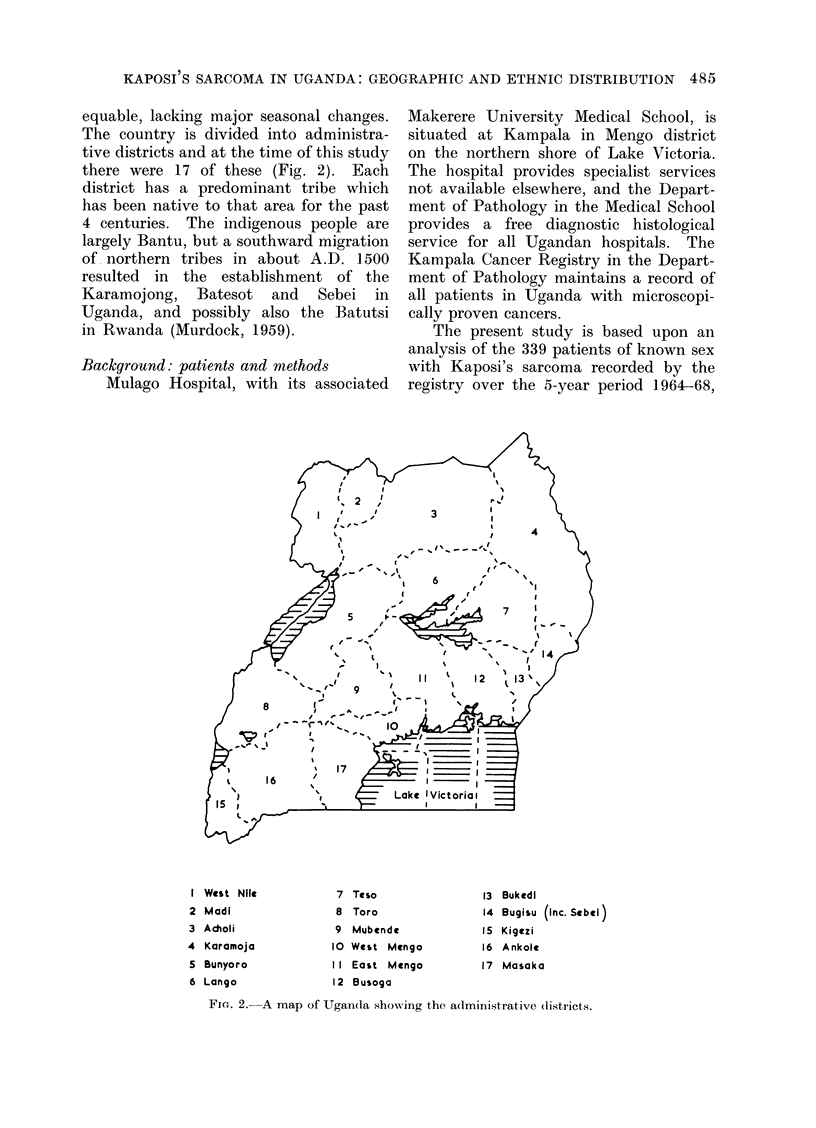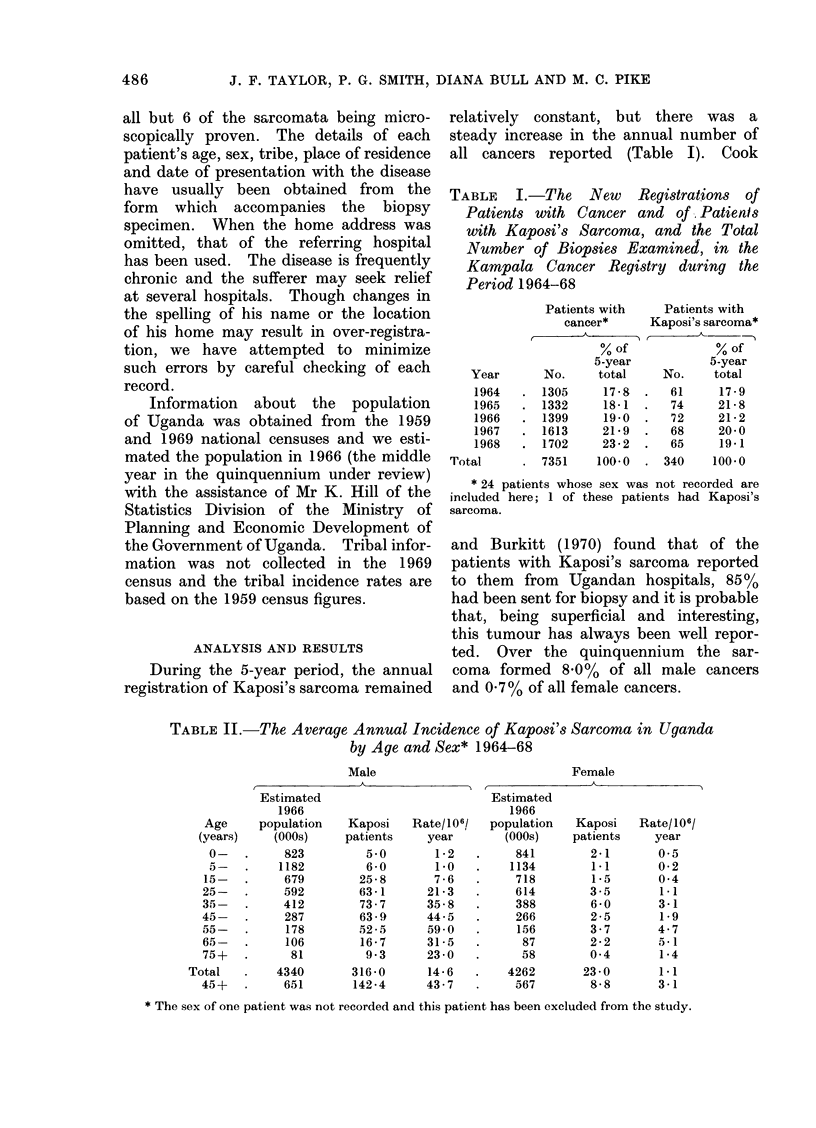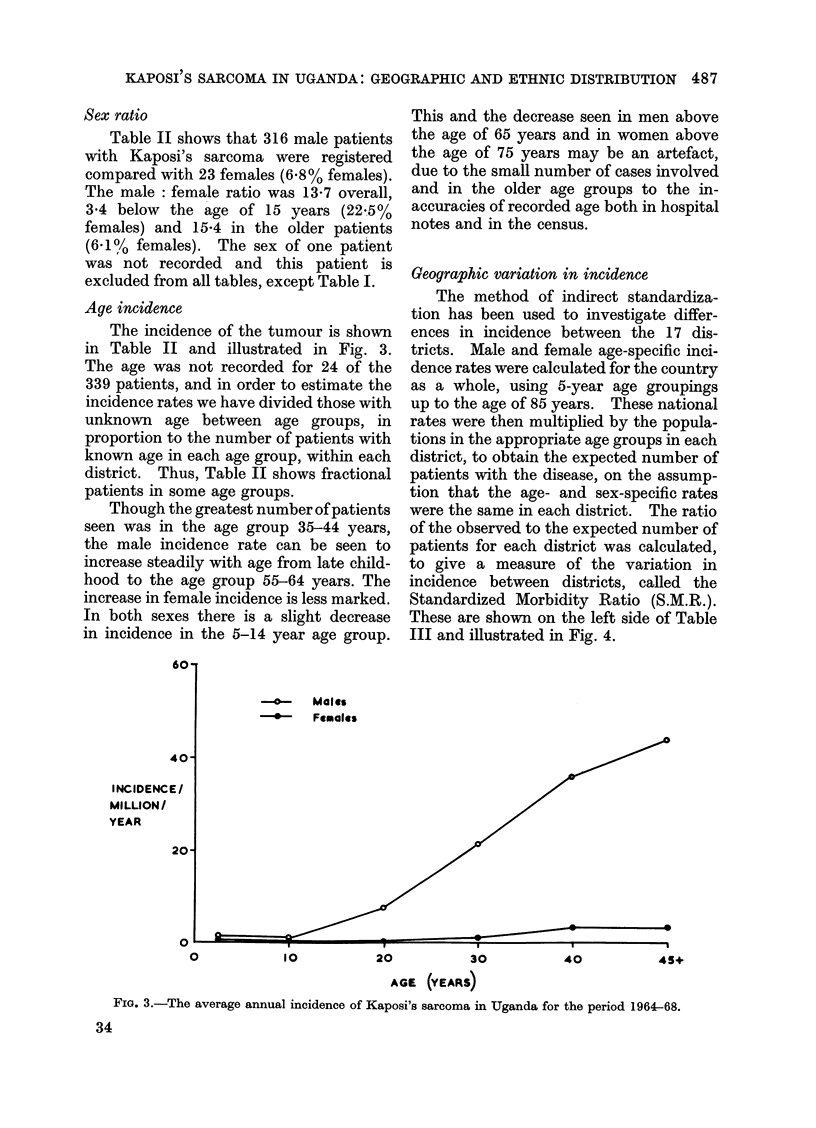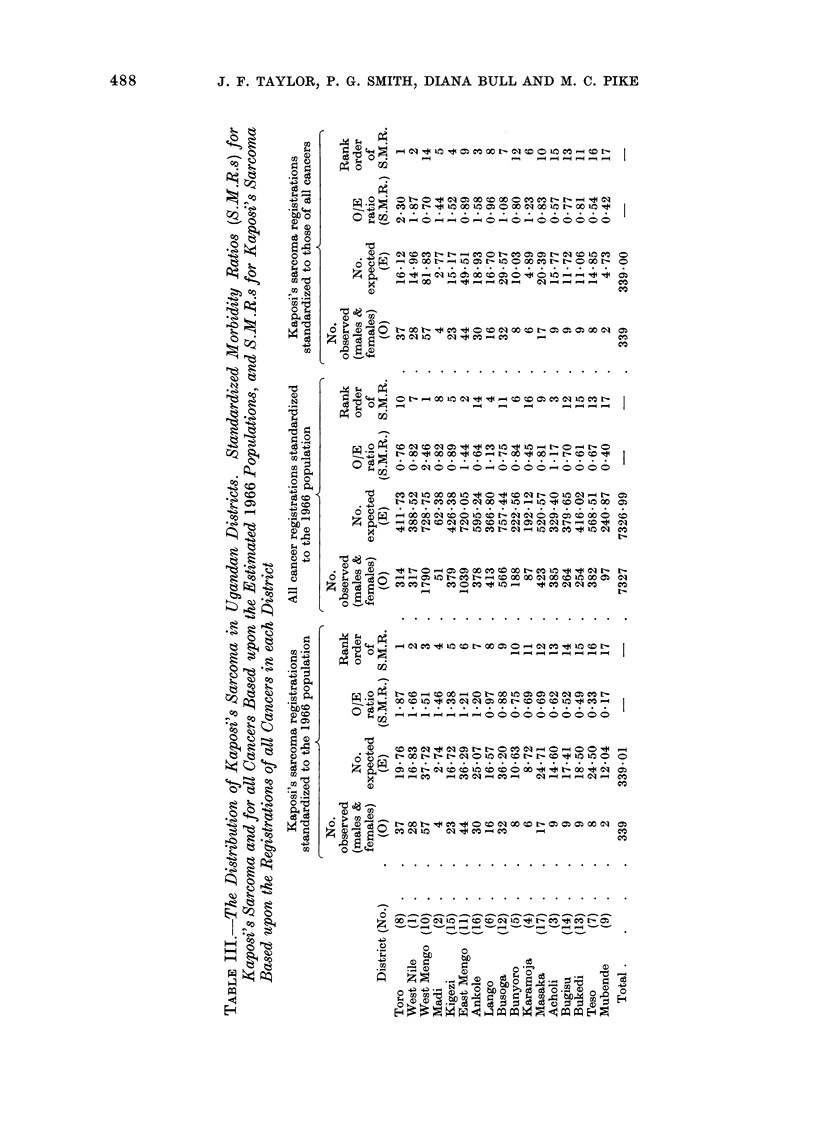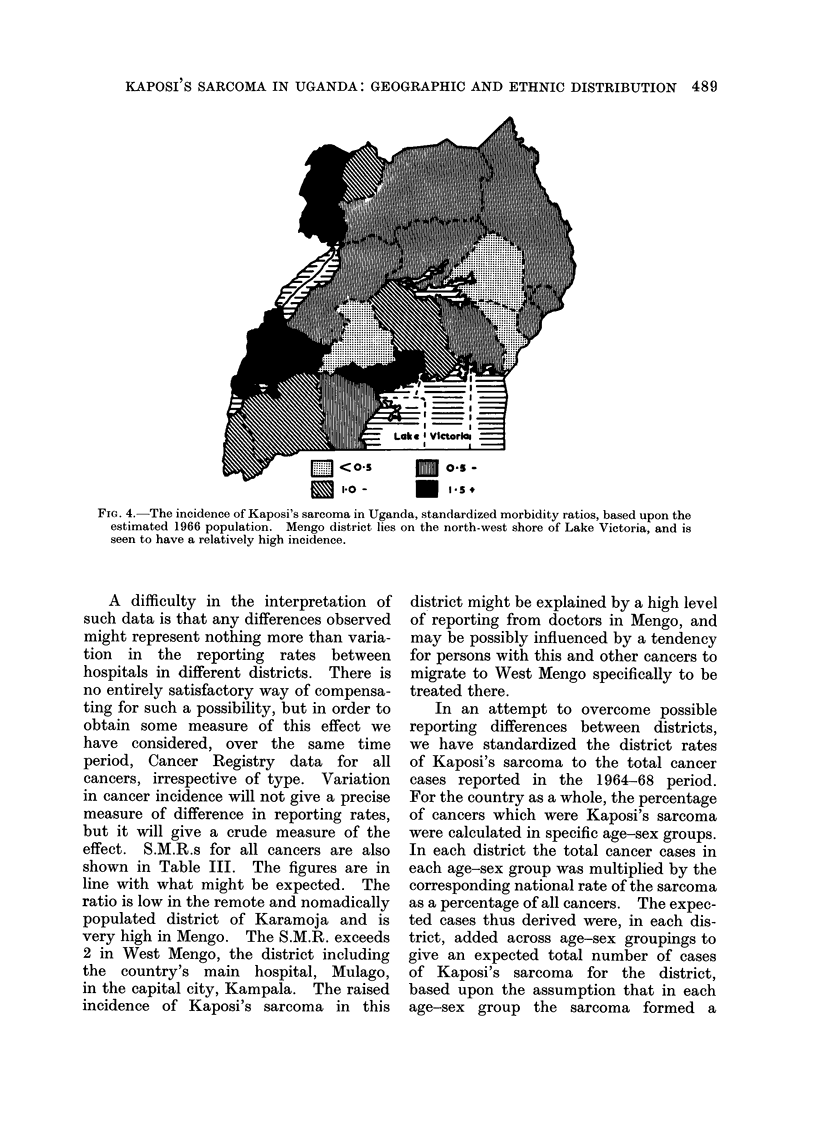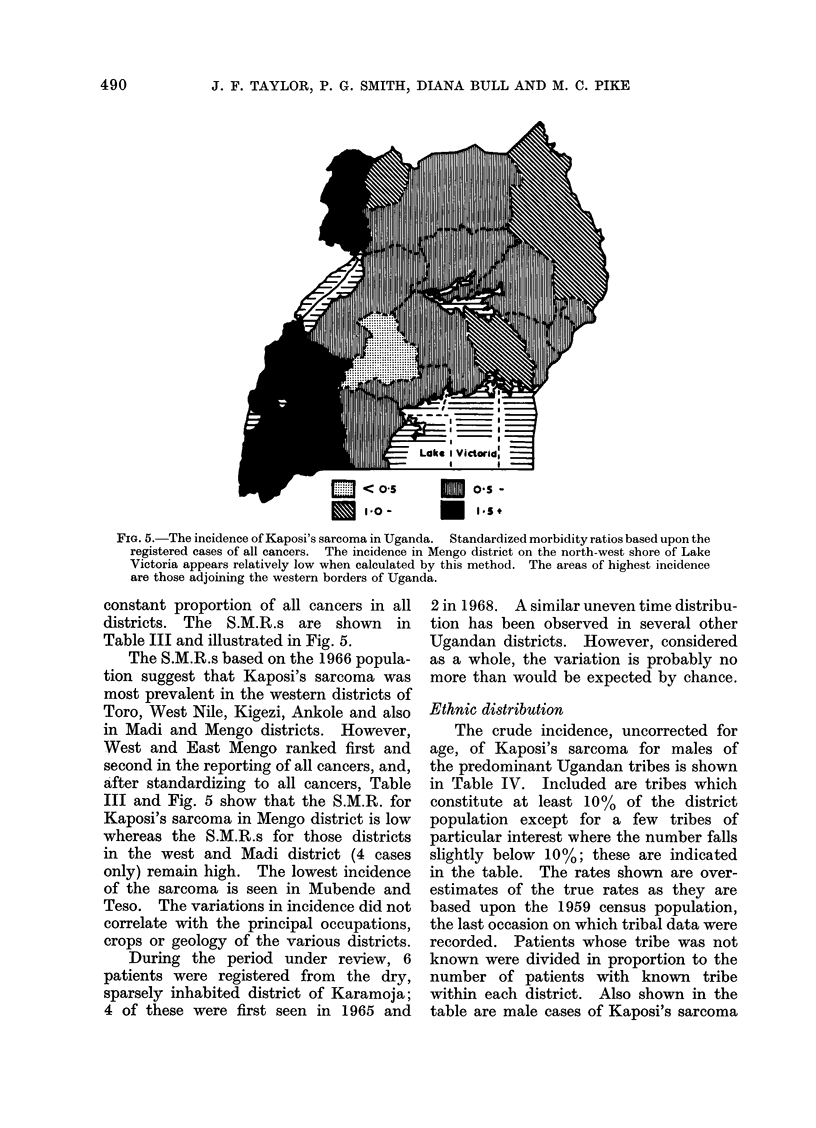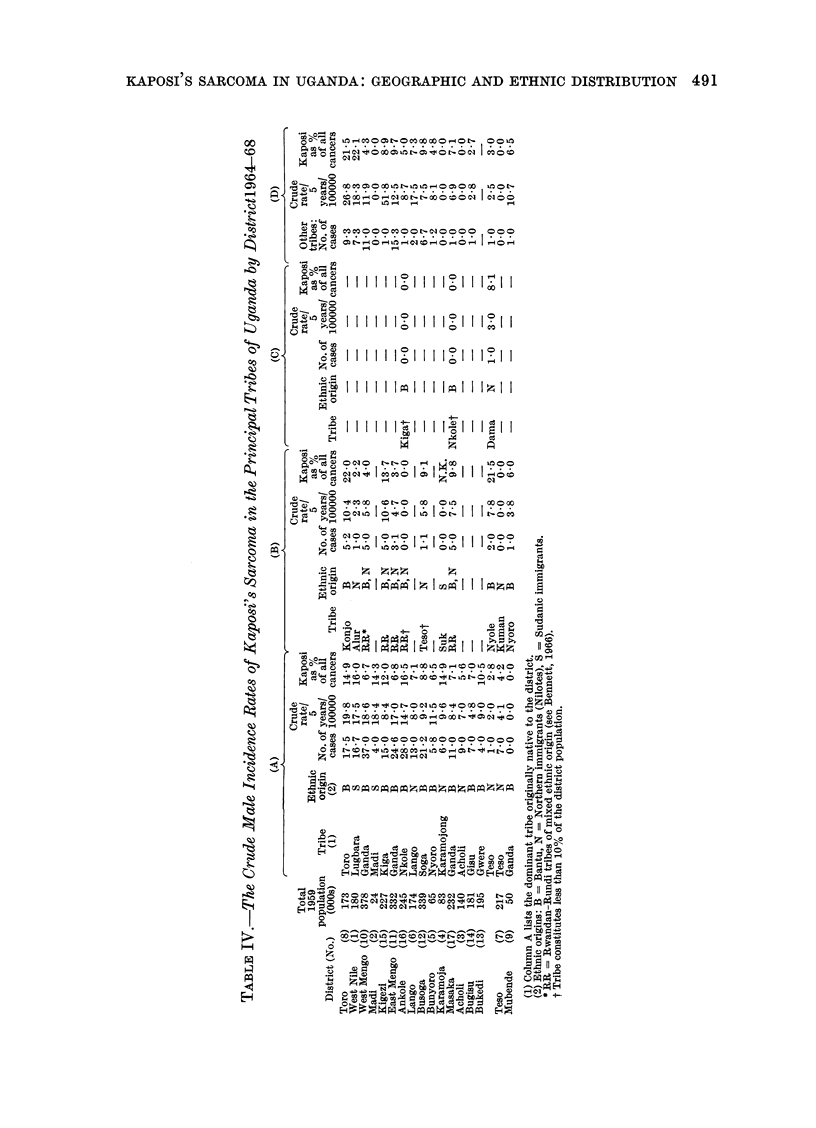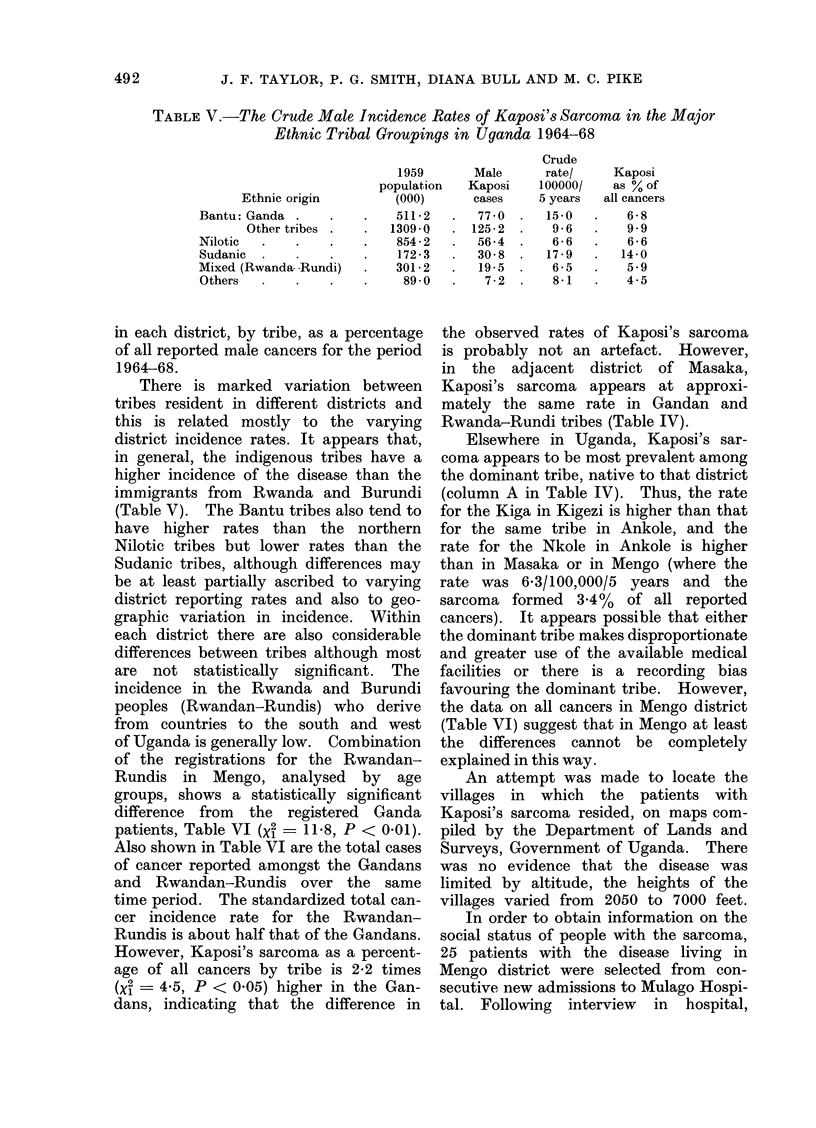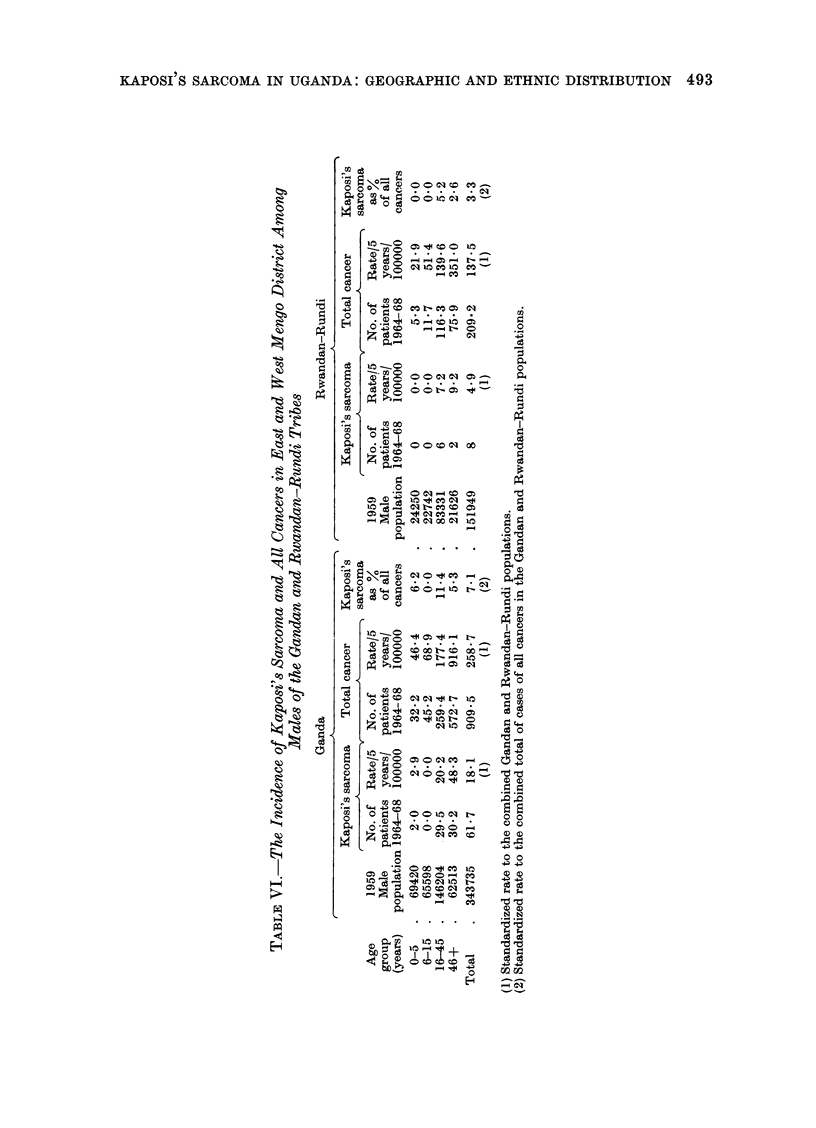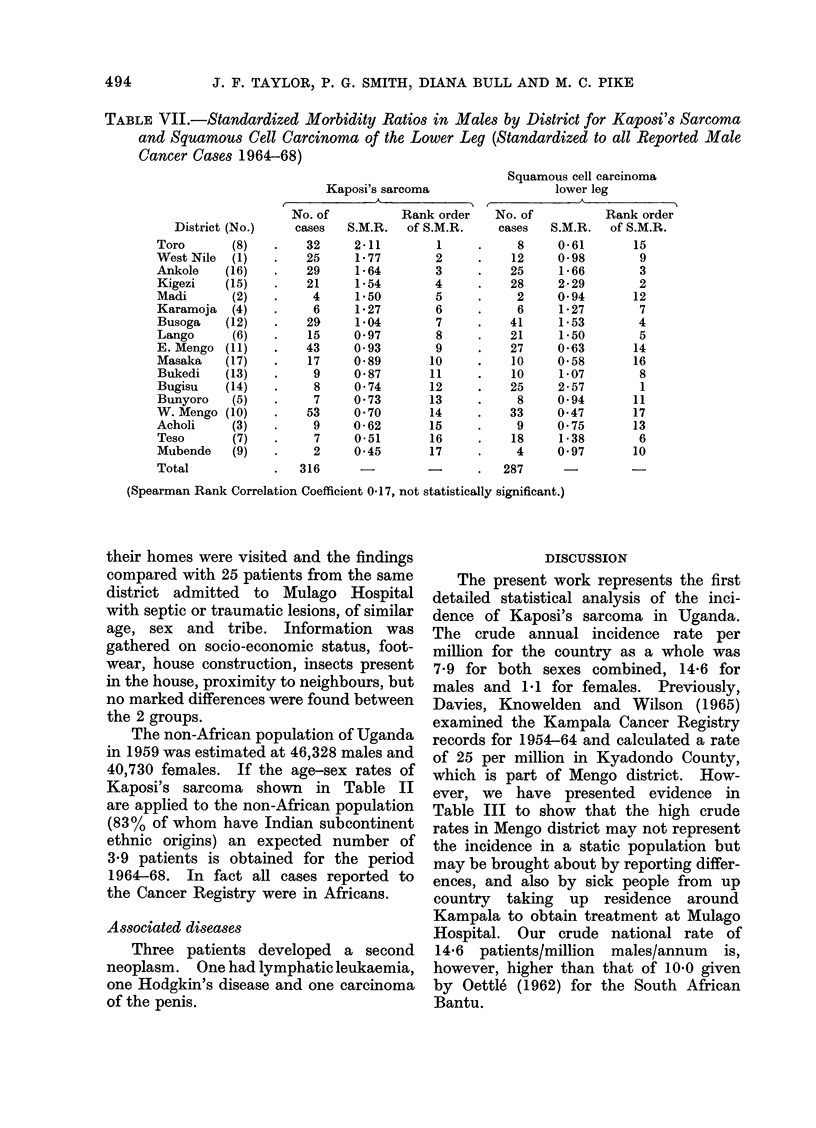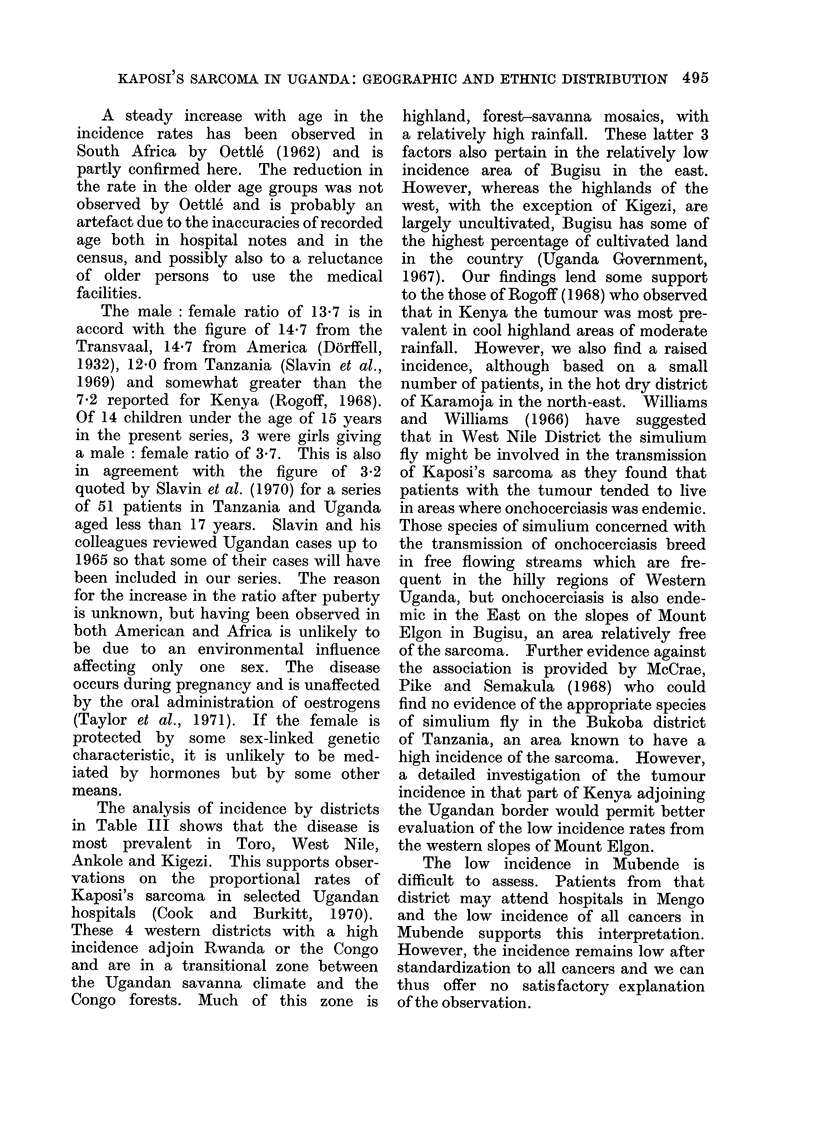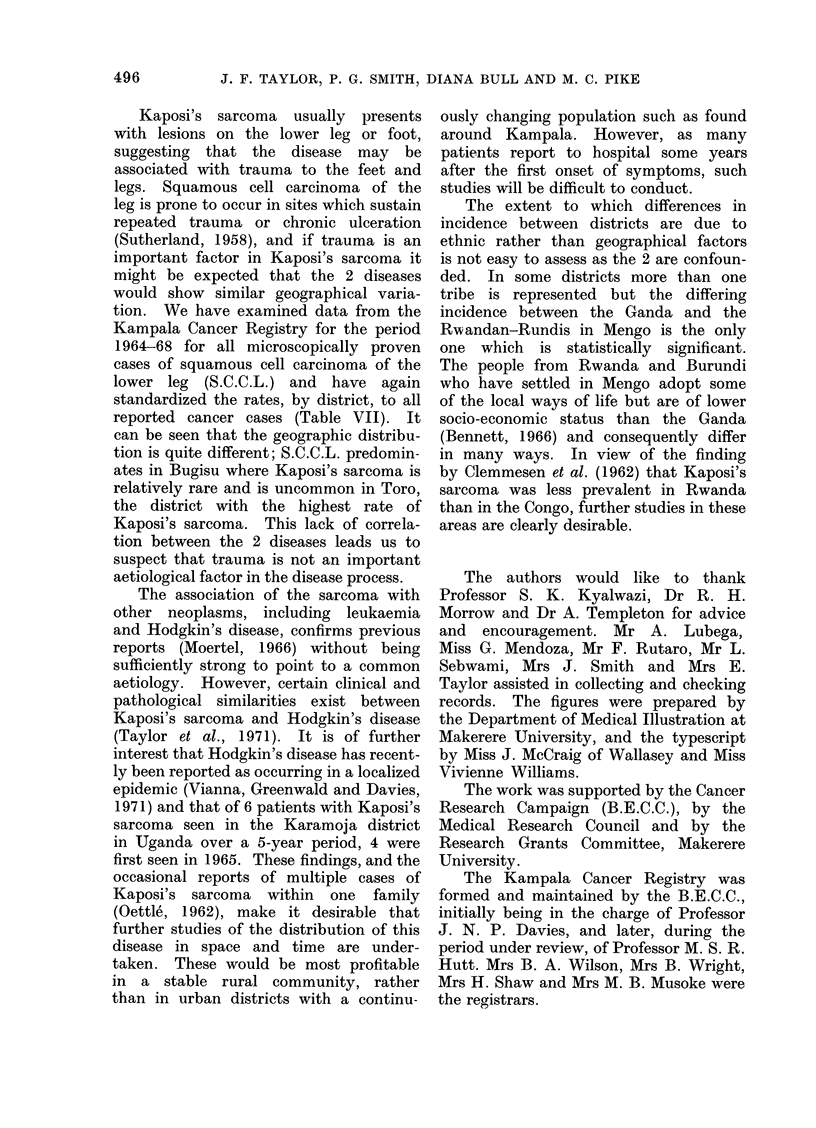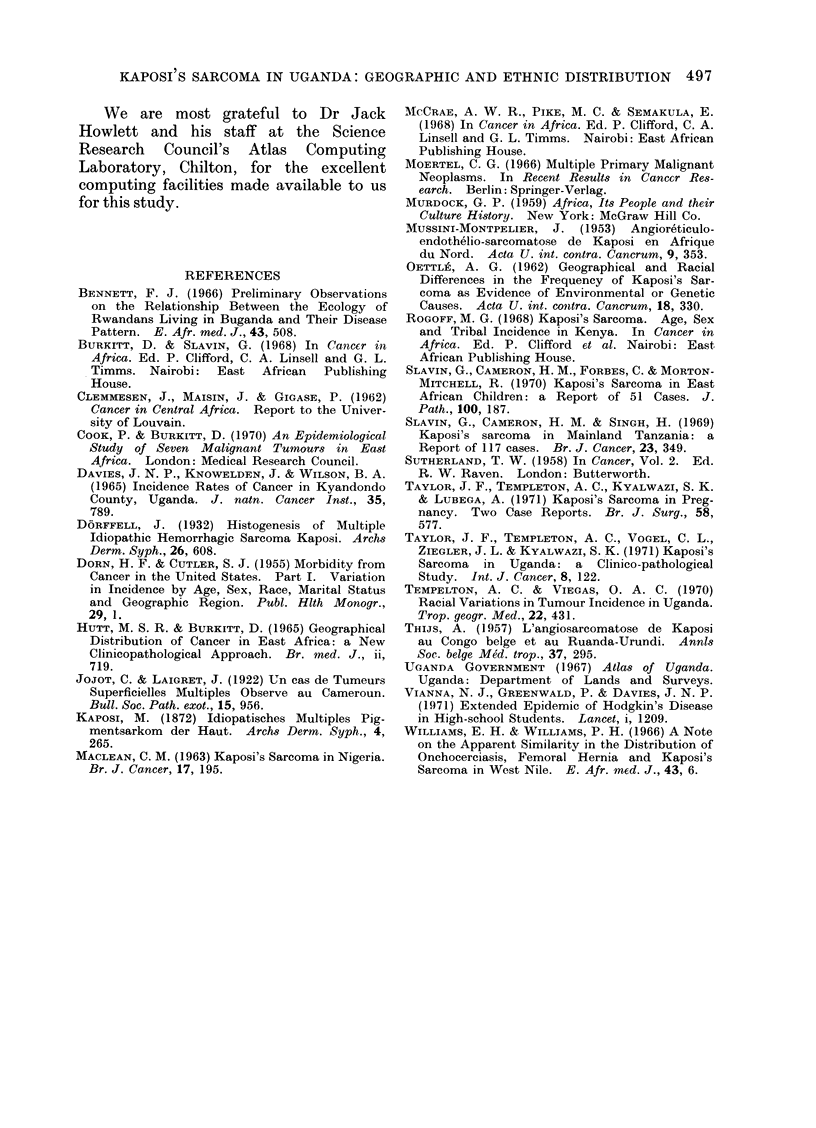# Kaposi's Sarcoma in Uganda: Geographic and Ethnic Distribution

**DOI:** 10.1038/bjc.1972.66

**Published:** 1972-12

**Authors:** J. F. Taylor, P. G. Smith, Diana Bull, M. C. Pike

## Abstract

Over the quinquennium 1964-68 the crude annual incidence of Kaposi's sarcoma in Uganda per million of the population was 7·9 overall, 14·6 for males and 1·1 for females. Statistical analysis indicates that the disease is most prevalent in highland areas to the west and among the indigenous Bantu tribes. There was no correlation with the distribution of squamous cell carcinoma of the lower leg, and Kaposi's sarcoma was not seen in an Indian or European during the period under review.


*Br. J. Cancer* (1972) **26**, 483.

### KAPOSI'S SARCOMA IN UGANDA: GEOGRAPHIC AND ETHNIC DISTRIBUTION

J. F. TAYLOR*, P. G. SMITH, DIANA BULL AND M. C. PIKE†

*From the Departments of Surgery and Preventive Medicine, Makerere University Medical School, P.O. Box 7072, Kampala, Uganda, and D.H.S.S. Cancer Epidemiology Unit, Regius Department of Medicine, 9 Keble Road, Oxford, U.K.*

Received 16 June 1972. Accepted 26 July 1972

**Summary.—Over the quinquennium 1964–68 the crude annual incidence of Kaposi's sarcoma in Uganda per million of the population was 7·9 overall, 14·6 for males and 1·1 for females. Statistical analysis indicates that the disease is most prevalent in highland areas to the west and among the indigenous Bantu tribes. There was no correlation with the distribution of squamous cell carcinoma of the lower leg, and Kaposi's sarcoma was not seen in an Indian or European during the period under review.**

NEARLY a century has passed since Moricz Kaposi (1872) described the multiple pigmented haemorrhagic sarcoma of skin now known as Kaposi's sarcoma. Early observations in Europe and America suggested that the disease was most prevalent in Russia, Poland and Northern Italy (Dörffell, 1932). However, subsequent to a report of an African case from the Cameroons (Jojot and Laigret, 1922) it has become clear that the disease is more prevalent in some parts of Africa than elsewhere. The reports from various African countries have been reviewed by Maclean (1963). Ratio studies, expressing the number of cases of Kaposi's sarcoma as a percentage of all cancers, suggest that the relative frequency is greatest in the N.E. Congo and in Rwanda and Burundi, diminishing radially in all directions (Oettlé, 1962). The decrease is, however, irregular. Thijs (1957) reported that the sarcoma formed 10·4% of 221 cancers sent for histology to Stanleyville from Rwanda between 1939 and 1955, but Clemmesen, Maisin and Gigase (1962) reported a ratio of only 0·9% in Rwanda and Burundi. A similar variable distribution has been reported from Tanzania (Burkitt and Slavin, 1968) and Kenya where Rogoff (1968) noted that the tribes with the highest incidence lived in high, cool country with a moderate rainfall. In Uganda, ratio studies (Cook and Burkitt, 1970; Hutt and Burkitt, 1965) have suggested that the disease is most prevalent in the west.

*Race and sex*

The crude incidence rate of the disease in Caucasian American males is 0·081/100,000, which is similar to the rate for the U.S. negro (0·103/100,000) (Dorn and Cutler, 1955) but in South Africa Oettlé (1962) observed that Kaposi's sarcoma was 10 times more common among the Bantu population than the white. To what extent a difference in environment or habit explains racial differences is uncertain. The majority of cases occur in men, and in East Africa less than 10% of adult patients are females (Slavin, Cameron and Singh, 1969), though below the age of 16 years this figure may be as high as 24% (Slavin *et al.*, 1970). However, among South

* Present address, Department of Orthopaedic Surgery, P.O. Box 147, Liverpool.
† Address for reprints, Dr M. C. Pike, D.H.S.S. Cancer Epidemiology Unit.

African and Algerian whites, females predominate in all age groups (Oettlé, 1962; Mussini-Montpelier, 1953). The female domestic environment differs from one country to another and is unlikely to be responsible for the predominant occurence of the tumour in males in all continents. It is possible that the X chromosomes carry a recessive gene capable of preventing initiation or maintenance of the disease though this has been argued against by Oettlé (1962) because of the low sex ratio in Durban Bantu and in South African and Algerian whites.

In the present work we describe an epidemiological analysis of the records for Kaposi's sarcoma in the Kampala Cancer Registry, Uganda.

*Site of the investigation*

Uganda lies at an altitude of about 4000 feet on the north shore of Lake Victoria. In the central regions the vegetation is mixed moist savanna and shrub woodlands. Mount Elgon lies on the Kenya border to the east, and further north the dry savanna plains of Karamoja adjoin the Sudan border. To the west there is a gradual increase in altitude to the mountains of Ankole and Kigezi, and the Ruwenzoris on the Congo border (Fig. 1). The climate is warm and

FIG. 1.—A relief map of Uganda. The areas of high altitude above 4000 feet should be compared with the high incidence areas of Kaposi's sarcoma seen in Fig. 4.

equable, lacking major seasonal changes. The country is divided into administrative districts and at the time of this study there were 17 of these (Fig. 2). Each district has a predominant tribe which has been native to that area for the past 4 centuries. The indigenous people are largely Bantu, but a southward migration of northern tribes in about A.D. 1500 resulted in the establishment of the Karamojong, Batesot and Sebei in Uganda, and possibly also the Batutsi in Rwanda (Murdock, 1959).

*Background: patients and methods*

Mulago Hospital, with its associated Makerere University Medical School, is situated at Kampala in Mengo district on the northern shore of Lake Victoria. The hospital provides specialist services not available elsewhere, and the Department of Pathology in the Medical School provides a free diagnostic histological service for all Ugandan hospitals. The Kampala Cancer Registry in the Department of Pathology maintains a record of all patients in Uganda with microscopically proven cancers.

The present study is based upon an analysis of the 339 patients of known sex with Kaposi's sarcoma recorded by the registry over the 5-year period 1964–68,

| | | |
|---|---|---|
| 1 West Nile | 7 Teso | 13 Bukedi |
| 2 Madi | 8 Toro | 14 Bugisu (inc. Sebei) |
| 3 Acholi | 9 Mubende | 15 Kigezi |
| 4 Karamoja | 10 West Mengo | 16 Ankole |
| 5 Bunyoro | 11 East Mengo | 17 Masaka |
| 6 Lango | 12 Busoga | |

FIG. 2.—A map of Uganda showing the administrative districts.

all but 6 of the sarcomata being microscopically proven. The details of each patient's age, sex, tribe, place of residence and date of presentation with the disease have usually been obtained from the form which accompanies the biopsy specimen. When the home address was omitted, that of the referring hospital has been used. The disease is frequently chronic and the sufferer may seek relief at several hospitals. Though changes in the spelling of his name or the location of his home may result in over-registration, we have attempted to minimize such errors by careful checking of each record.

Information about the population of Uganda was obtained from the 1959 and 1969 national censuses and we estimated the population in 1966 (the middle year in the quinquennium under review) with the assistance of Mr K. Hill of the Statistics Division of the Ministry of Planning and Economic Development of the Government of Uganda. Tribal information was not collected in the 1969 census and the tribal incidence rates are based on the 1959 census figures.

### ANALYSIS AND RESULTS

During the 5-year period, the annual registration of Kaposi's sarcoma remained relatively constant, but there was a steady increase in the annual number of all cancers reported (Table I). Cook and Burkitt (1970) found that of the patients with Kaposi's sarcoma reported to them from Ugandan hospitals, 85% had been sent for biopsy and it is probable that, being superficial and interesting, this tumour has always been well reported. Over the quinquennium the sarcoma formed 8·0% of all male cancers and 0·7% of all female cancers.

TABLE I.—*The New Registrations of Patients with Cancer and of Patients with Kaposi's Sarcoma, and the Total Number of Biopsies Examined, in the Kampala Cancer Registry during the Period* 1964–68

| | Patients with cancer* | | Patients with Kaposi's sarcoma* | |
|---|---|---|---|---|
| Year | No. | % of 5-year total | No. | % of 5-year total |
| 1964 | 1305 | 17·8 | 61 | 17·9 |
| 1965 | 1332 | 18·1 | 74 | 21·8 |
| 1966 | 1399 | 19·0 | 72 | 21·2 |
| 1967 | 1613 | 21·9 | 68 | 20·0 |
| 1968 | 1702 | 23·2 | 65 | 19·1 |
| Total | 7351 | 100·0 | 340 | 100·0 |

* 24 patients whose sex was not recorded are included here; 1 of these patients had Kaposi's sarcoma.

TABLE II.—*The Average Annual Incidence of Kaposi's Sarcoma in Uganda by Age and Sex** 1964–68

| | Male | | | Female | | |
|---|---|---|---|---|---|---|
| Age (years) | Estimated 1966 population (000s) | Kaposi patients | Rate/$10^6$/year | Estimated 1966 population (000s) | Kaposi patients | Rate/$10^6$/year |
| 0– | 823 | 5·0 | 1·2 | 841 | 2·1 | 0·5 |
| 5– | 1182 | 6·0 | 1·0 | 1134 | 1·1 | 0·2 |
| 15– | 679 | 25·8 | 7·6 | 718 | 1·5 | 0·4 |
| 25– | 592 | 63·1 | 21·3 | 614 | 3·5 | 1·1 |
| 35– | 412 | 73·7 | 35·8 | 388 | 6·0 | 3·1 |
| 45– | 287 | 63·9 | 44·5 | 266 | 2·5 | 1·9 |
| 55– | 178 | 52·5 | 59·0 | 156 | 3·7 | 4·7 |
| 65– | 106 | 16·7 | 31·5 | 87 | 2·2 | 5·1 |
| 75+ | 81 | 9·3 | 23·0 | 58 | 0·4 | 1·4 |
| Total | 4340 | 316·0 | 14·6 | 4262 | 23·0 | 1·1 |
| 45+ | 651 | 142·4 | 43·7 | 567 | 8·8 | 3·1 |

* The sex of one patient was not recorded and this patient has been excluded from the study.

*Sex ratio*

Table II shows that 316 male patients with Kaposi's sarcoma were registered compared with 23 females (6·8% females). The male : female ratio was 13·7 overall, 3·4 below the age of 15 years (22·5% females) and 15·4 in the older patients (6·1% females). The sex of one patient was not recorded and this patient is excluded from all tables, except Table I.

*Age incidence*

The incidence of the tumour is shown in Table II and illustrated in Fig. 3. The age was not recorded for 24 of the 339 patients, and in order to estimate the incidence rates we have divided those with unknown age between age groups, in proportion to the number of patients with known age in each age group, within each district. Thus, Table II shows fractional patients in some age groups.

Though the greatest number of patients seen was in the age group 35–44 years, the male incidence rate can be seen to increase steadily with age from late childhood to the age group 55–64 years. The increase in female incidence is less marked. In both sexes there is a slight decrease in incidence in the 5–14 year age group. This and the decrease seen in men above the age of 65 years and in women above the age of 75 years may be an artefact, due to the small number of cases involved and in the older age groups to the inaccuracies of recorded age both in hospital notes and in the census.

*Geographic variation in incidence*

The method of indirect standardization has been used to investigate differences in incidence between the 17 districts. Male and female age-specific incidence rates were calculated for the country as a whole, using 5-year age groupings up to the age of 85 years. These national rates were then multiplied by the populations in the appropriate age groups in each district, to obtain the expected number of patients with the disease, on the assumption that the age- and sex-specific rates were the same in each district. The ratio of the observed to the expected number of patients for each district was calculated, to give a measure of the variation in incidence between districts, called the Standardized Morbidity Ratio (S.M.R.). These are shown on the left side of Table III and illustrated in Fig. 4.

FIG. 3.—The average annual incidence of Kaposi's sarcoma in Uganda for the period 1964–68.

FIG. 4.—The incidence of Kaposi's sarcoma in Uganda, standardized morbidity ratios, based upon the estimated 1966 population. Mengo district lies on the north-west shore of Lake Victoria, and is seen to have a relatively high incidence.

A difficulty in the interpretation of such data is that any differences observed might represent nothing more than variation in the reporting rates between hospitals in different districts. There is no entirely satisfactory way of compensating for such a possibility, but in order to obtain some measure of this effect we have considered, over the same time period, Cancer Registry data for all cancers, irrespective of type. Variation in cancer incidence will not give a precise measure of difference in reporting rates, but it will give a crude measure of the effect. S.M.R.s for all cancers are also shown in Table III. The figures are in line with what might be expected. The ratio is low in the remote and nomadically populated district of Karamoja and is very high in Mengo. The S.M.R. exceeds 2 in West Mengo, the district including the country's main hospital, Mulago, in the capital city, Kampala. The raised incidence of Kaposi's sarcoma in this district might be explained by a high level of reporting from doctors in Mengo, and may be possibly influenced by a tendency for persons with this and other cancers to migrate to West Mengo specifically to be treated there.

In an attempt to overcome possible reporting differences between districts, we have standardized the district rates of Kaposi's sarcoma to the total cancer cases reported in the 1964–68 period. For the country as a whole, the percentage of cancers which were Kaposi's sarcoma were calculated in specific age–sex groups. In each district the total cancer cases in each age–sex group was multiplied by the corresponding national rate of the sarcoma as a percentage of all cancers. The expected cases thus derived were, in each district, added across age–sex groupings to give an expected total number of cases of Kaposi's sarcoma for the district, based upon the assumption that in each age–sex group the sarcoma formed a

FIG. 5.—The incidence of Kaposi's sarcoma in Uganda. Standardized morbidity ratios based upon the registered cases of all cancers. The incidence in Mengo district on the north-west shore of Lake Victoria appears relatively low when calculated by this method. The areas of highest incidence are those adjoining the western borders of Uganda.

constant proportion of all cancers in all districts. The S.M.R.s are shown in Table III and illustrated in Fig. 5.

The S.M.R.s based on the 1966 population suggest that Kaposi's sarcoma was most prevalent in the western districts of Toro, West Nile, Kigezi, Ankole and also in Madi and Mengo districts. However, West and East Mengo ranked first and second in the reporting of all cancers, and, after standardizing to all cancers, Table III and Fig. 5 show that the S.M.R. for Kaposi's sarcoma in Mengo district is low whereas the S.M.R.s for those districts in the west and Madi district (4 cases only) remain high. The lowest incidence of the sarcoma is seen in Mubende and Teso. The variations in incidence did not correlate with the principal occupations, crops or geology of the various districts.

During the period under review, 6 patients were registered from the dry, sparsely inhabited district of Karamoja; 4 of these were first seen in 1965 and 2 in 1968. A similar uneven time distribution has been observed in several other Ugandan districts. However, considered as a whole, the variation is probably no more than would be expected by chance.

*Ethnic distribution*

The crude incidence, uncorrected for age, of Kaposi's sarcoma for males of the predominant Ugandan tribes is shown in Table IV. Included are tribes which constitute at least 10% of the district population except for a few tribes of particular interest where the number falls slightly below 10%; these are indicated in the table. The rates shown are overestimates of the true rates as they are based upon the 1959 census population, the last occasion on which tribal data were recorded. Patients whose tribe was not known were divided in proportion to the number of patients with known tribe within each district. Also shown in the table are male cases of Kaposi's sarcoma

TABLE V.—*The Crude Male Incidence Rates of Kaposi's Sarcoma in the Major Ethnic Tribal Groupings in Uganda* 1964–68

| Ethnic origin | 1959 population (000) | Male Kaposi cases | Crude rate/ 100000/ 5 years | Kaposi as % of all cancers |
|---|---|---|---|---|
| Bantu: Ganda | 511·2 | 77·0 | 15·0 | 6·8 |
| Other tribes | 1309·0 | 125·2 | 9·6 | 9·9 |
| Nilotic | 854·2 | 56·4 | 6·6 | 6·6 |
| Sudanic | 172·3 | 30·8 | 17·9 | 14·0 |
| Mixed (Rwanda–Rundi) | 301·2 | 19·5 | 6·5 | 5·9 |
| Others | 89·0 | 7·2 | 8·1 | 4·5 |

in each district, by tribe, as a percentage of all reported male cancers for the period 1964–68.

There is marked variation between tribes resident in different districts and this is related mostly to the varying district incidence rates. It appears that, in general, the indigenous tribes have a higher incidence of the disease than the immigrants from Rwanda and Burundi (Table V). The Bantu tribes also tend to have higher rates than the northern Nilotic tribes but lower rates than the Sudanic tribes, although differences may be at least partially ascribed to varying district reporting rates and also to geographic variation in incidence. Within each district there are also considerable differences between tribes although most are not statistically significant. The incidence in the Rwanda and Burundi peoples (Rwandan–Rundis) who derive from countries to the south and west of Uganda is generally low. Combination of the registrations for the Rwandan–Rundis in Mengo, analysed by age groups, shows a statistically significant difference from the registered Ganda patients, Table VI ($\chi_1^2 = 11{\cdot}8$, $P < 0{\cdot}01$). Also shown in Table VI are the total cases of cancer reported amongst the Gandans and Rwandan–Rundis over the same time period. The standardized total cancer incidence rate for the Rwandan–Rundis is about half that of the Gandans. However, Kaposi's sarcoma as a percentage of all cancers by tribe is 2·2 times ($\chi_1^2 = 4{\cdot}5$, $P < 0{\cdot}05$) higher in the Gandans, indicating that the difference in the observed rates of Kaposi's sarcoma is probably not an artefact. However, in the adjacent district of Masaka, Kaposi's sarcoma appears at approximately the same rate in Gandan and Rwanda–Rundi tribes (Table IV).

Elsewhere in Uganda, Kaposi's sarcoma appears to be most prevalent among the dominant tribe, native to that district (column A in Table IV). Thus, the rate for the Kiga in Kigezi is higher than that for the same tribe in Ankole, and the rate for the Nkole in Ankole is higher than in Masaka or in Mengo (where the rate was 6·3/100,000/5 years and the sarcoma formed 3·4% of all reported cancers). It appears possible that either the dominant tribe makes disproportionate and greater use of the available medical facilities or there is a recording bias favouring the dominant tribe. However, the data on all cancers in Mengo district (Table VI) suggest that in Mengo at least the differences cannot be completely explained in this way.

An attempt was made to locate the villages in which the patients with Kaposi's sarcoma resided, on maps compiled by the Department of Lands and Surveys, Government of Uganda. There was no evidence that the disease was limited by altitude, the heights of the villages varied from 2050 to 7000 feet.

In order to obtain information on the social status of people with the sarcoma, 25 patients with the disease living in Mengo district were selected from consecutive new admissions to Mulago Hospital. Following interview in hospital,

TABLE VII.—*Standardized Morbidity Ratios in Males by District for Kaposi's Sarcoma and Squamous Cell Carcinoma of the Lower Leg* (*Standardized to all Reported Male Cancer Cases* 1964–68)

| District | (No.) | Kaposi's sarcoma No. of cases | Kaposi's sarcoma S.M.R. | Kaposi's sarcoma Rank order of S.M.R. | Squamous cell carcinoma lower leg No. of cases | Squamous cell carcinoma lower leg S.M.R. | Squamous cell carcinoma lower leg Rank order of S.M.R. |
|---|---|---|---|---|---|---|---|
| Toro | (8) | 32 | 2·11 | 1 | 8 | 0·61 | 15 |
| West Nile | (1) | 25 | 1·77 | 2 | 12 | 0·98 | 9 |
| Ankole | (16) | 29 | 1·64 | 3 | 25 | 1·66 | 3 |
| Kigezi | (15) | 21 | 1·54 | 4 | 28 | 2·29 | 2 |
| Madi | (2) | 4 | 1·50 | 5 | 2 | 0·94 | 12 |
| Karamoja | (4) | 6 | 1·27 | 6 | 6 | 1·27 | 7 |
| Busoga | (12) | 29 | 1·04 | 7 | 41 | 1·53 | 4 |
| Lango | (6) | 15 | 0·97 | 8 | 21 | 1·50 | 5 |
| E. Mengo | (11) | 43 | 0·93 | 9 | 27 | 0·63 | 14 |
| Masaka | (17) | 17 | 0·89 | 10 | 10 | 0·58 | 16 |
| Bukedi | (13) | 9 | 0·87 | 11 | 10 | 1·07 | 8 |
| Bugisu | (14) | 8 | 0·74 | 12 | 25 | 2·57 | 1 |
| Bunyoro | (5) | 7 | 0·73 | 13 | 8 | 0·94 | 11 |
| W. Mengo | (10) | 53 | 0·70 | 14 | 33 | 0·47 | 17 |
| Acholi | (3) | 9 | 0·62 | 15 | 9 | 0·75 | 13 |
| Teso | (7) | 7 | 0·51 | 16 | 18 | 1·38 | 6 |
| Mubende | (9) | 2 | 0·45 | 17 | 4 | 0·97 | 10 |
| Total | | 316 | — | — | 287 | — | — |

(Spearman Rank Correlation Coefficient 0·17, not statistically significant.)

their homes were visited and the findings compared with 25 patients from the same district admitted to Mulago Hospital with septic or traumatic lesions, of similar age, sex and tribe. Information was gathered on socio-economic status, footwear, house construction, insects present in the house, proximity to neighbours, but no marked differences were found between the 2 groups.

The non-African population of Uganda in 1959 was estimated at 46,328 males and 40,730 females. If the age–sex rates of Kaposi's sarcoma shown in Table II are applied to the non-African population (83% of whom have Indian subcontinent ethnic origins) an expected number of 3·9 patients is obtained for the period 1964–68. In fact all cases reported to the Cancer Registry were in Africans.

*Associated diseases*

Three patients developed a second neoplasm. One had lymphatic leukaemia, one Hodgkin's disease and one carcinoma of the penis.

### DISCUSSION

The present work represents the first detailed statistical analysis of the incidence of Kaposi's sarcoma in Uganda. The crude annual incidence rate per million for the country as a whole was 7·9 for both sexes combined, 14·6 for males and 1·1 for females. Previously, Davies, Knowelden and Wilson (1965) examined the Kampala Cancer Registry records for 1954–64 and calculated a rate of 25 per million in Kyadondo County, which is part of Mengo district. However, we have presented evidence in Table III to show that the high crude rates in Mengo district may not represent the incidence in a static population but may be brought about by reporting differences, and also by sick people from up country taking up residence around Kampala to obtain treatment at Mulago Hospital. Our crude national rate of 14·6 patients/million males/annum is, however, higher than that of 10·0 given by Oettlé (1962) for the South African Bantu.

A steady increase with age in the incidence rates has been observed in South Africa by Oettlé (1962) and is partly confirmed here. The reduction in the rate in the older age groups was not observed by Oettlé and is probably an artefact due to the inaccuracies of recorded age both in hospital notes and in the census, and possibly also to a reluctance of older persons to use the medical facilities.

The male : female ratio of 13·7 is in accord with the figure of 14·7 from the Transvaal, 14·7 from America (Dörffell, 1932), 12·0 from Tanzania (Slavin *et al.*, 1969) and somewhat greater than the 7·2 reported for Kenya (Rogoff, 1968). Of 14 children under the age of 15 years in the present series, 3 were girls giving a male : female ratio of 3·7. This is also in agreement with the figure of 3·2 quoted by Slavin *et al.* (1970) for a series of 51 patients in Tanzania and Uganda aged less than 17 years. Slavin and his colleagues reviewed Ugandan cases up to 1965 so that some of their cases will have been included in our series. The reason for the increase in the ratio after puberty is unknown, but having been observed in both American and Africa is unlikely to be due to an environmental influence affecting only one sex. The disease occurs during pregnancy and is unaffected by the oral administration of oestrogens (Taylor *et al.*, 1971). If the female is protected by some sex-linked genetic characteristic, it is unlikely to be mediated by hormones but by some other means.

The analysis of incidence by districts in Table III shows that the disease is most prevalent in Toro, West Nile, Ankole and Kigezi. This supports observations on the proportional rates of Kaposi's sarcoma in selected Ugandan hospitals (Cook and Burkitt, 1970). These 4 western districts with a high incidence adjoin Rwanda or the Congo and are in a transitional zone between the Ugandan savanna climate and the Congo forests. Much of this zone is highland, forest–savanna mosaics, with a relatively high rainfall. These latter 3 factors also pertain in the relatively low incidence area of Bugisu in the east. However, whereas the highlands of the west, with the exception of Kigezi, are largely uncultivated, Bugisu has some of the highest percentage of cultivated land in the country (Uganda Government, 1967). Our findings lend some support to the those of Rogoff (1968) who observed that in Kenya the tumour was most prevalent in cool highland areas of moderate rainfall. However, we also find a raised incidence, although based on a small number of patients, in the hot dry district of Karamoja in the north-east. Williams and Williams (1966) have suggested that in West Nile District the simulium fly might be involved in the transmission of Kaposi's sarcoma as they found that patients with the tumour tended to live in areas where onchocerciasis was endemic. Those species of simulium concerned with the transmission of onchocerciasis breed in free flowing streams which are frequent in the hilly regions of Western Uganda, but onchocerciasis is also endemic in the East on the slopes of Mount Elgon in Bugisu, an area relatively free of the sarcoma. Further evidence against the association is provided by McCrae, Pike and Semakula (1968) who could find no evidence of the appropriate species of simulium fly in the Bukoba district of Tanzania, an area known to have a high incidence of the sarcoma. However, a detailed investigation of the tumour incidence in that part of Kenya adjoining the Ugandan border would permit better evaluation of the low incidence rates from the western slopes of Mount Elgon.

The low incidence in Mubende is difficult to assess. Patients from that district may attend hospitals in Mengo and the low incidence of all cancers in Mubende supports this interpretation. However, the incidence remains low after standardization to all cancers and we can thus offer no satisfactory explanation of the observation.

Kaposi's sarcoma usually presents with lesions on the lower leg or foot, suggesting that the disease may be associated with trauma to the feet and legs. Squamous cell carcinoma of the leg is prone to occur in sites which sustain repeated trauma or chronic ulceration (Sutherland, 1958), and if trauma is an important factor in Kaposi's sarcoma it might be expected that the 2 diseases would show similar geographical variation. We have examined data from the Kampala Cancer Registry for the period 1964–68 for all microscopically proven cases of squamous cell carcinoma of the lower leg (S.C.C.L.) and have again standardized the rates, by district, to all reported cancer cases (Table VII). It can be seen that the geographic distribution is quite different; S.C.C.L. predominates in Bugisu where Kaposi's sarcoma is relatively rare and is uncommon in Toro, the district with the highest rate of Kaposi's sarcoma. This lack of correlation between the 2 diseases leads us to suspect that trauma is not an important aetiological factor in the disease process.

The association of the sarcoma with other neoplasms, including leukaemia and Hodgkin's disease, confirms previous reports (Moertel, 1966) without being sufficiently strong to point to a common aetiology. However, certain clinical and pathological similarities exist between Kaposi's sarcoma and Hodgkin's disease (Taylor *et al.*, 1971). It is of further interest that Hodgkin's disease has recently been reported as occurring in a localized epidemic (Vianna, Greenwald and Davies, 1971) and that of 6 patients with Kaposi's sarcoma seen in the Karamoja district in Uganda over a 5-year period, 4 were first seen in 1965. These findings, and the occasional reports of multiple cases of Kaposi's sarcoma within one family (Oettlé, 1962), make it desirable that further studies of the distribution of this disease in space and time are undertaken. These would be most profitable in a stable rural community, rather than in urban districts with a continuously changing population such as found around Kampala. However, as many patients report to hospital some years after the first onset of symptoms, such studies will be difficult to conduct.

The extent to which differences in incidence between districts are due to ethnic rather than geographical factors is not easy to assess as the 2 are confounded. In some districts more than one tribe is represented but the differing incidence between the Ganda and the Rwandan–Rundis in Mengo is the only one which is statistically significant. The people from Rwanda and Burundi who have settled in Mengo adopt some of the local ways of life but are of lower socio-economic status than the Ganda (Bennett, 1966) and consequently differ in many ways. In view of the finding by Clemmesen *et al.* (1962) that Kaposi's sarcoma was less prevalent in Rwanda than in the Congo, further studies in these areas are clearly desirable.

The authors would like to thank Professor S. K. Kyalwazi, Dr R. H. Morrow and Dr A. Templeton for advice and encouragement. Mr A. Lubega, Miss G. Mendoza, Mr F. Rutaro, Mr L. Sebwami, Mrs J. Smith and Mrs E. Taylor assisted in collecting and checking records. The figures were prepared by the Department of Medical Illustration at Makerere University, and the typescript by Miss J. McCraig of Wallasey and Miss Vivienne Williams.

The work was supported by the Cancer Research Campaign (B.E.C.C.), by the Medical Research Council and by the Research Grants Committee, Makerere University.

The Kampala Cancer Registry was formed and maintained by the B.E.C.C., initially being in the charge of Professor J. N. P. Davies, and later, during the period under review, of Professor M. S. R. Hutt. Mrs B. A. Wilson, Mrs B. Wright, Mrs H. Shaw and Mrs M. B. Musoke were the registrars.

We are most grateful to Dr Jack Howlett and his staff at the Science Research Council's Atlas Computing Laboratory, Chilton, for the excellent computing facilities made available to us for this study.

### REFERENCES

BENNETT, F. J. (1966) Preliminary Observations on the Relationship Between the Ecology of Rwandans Living in Buganda and Their Disease Pattern. *E. Afr. med. J.*, **43**, 508.

BURKITT, D. & SLAVIN, G. (1968) In *Cancer in Africa*. Ed. P. Clifford, C. A. Linsell and G. L. Timms. Nairobi: East African Publishing House.

CLEMMESEN, J., MAISIN, J. & GIGASE, P. (1962) *Cancer in Central Africa*. Report to the University of Louvain.

COOK, P. & BURKITT, D. (1970) *An Epidemiological Study of Seven Malignant Tumours in East Africa*. London: Medical Research Council.

DAVIES, J. N. P., KNOWELDEN, J. & WILSON, B. A. (1965) Incidence Rates of Cancer in Kyandondo County, Uganda. *J. natn. Cancer Inst.*, **35**, 789.

DÖRFFELL, J. (1932) Histogenesis of Multiple Idiopathic Hemorrhagic Sarcoma Kaposi. *Archs Derm. Syph.*, **26**, 608.

DORN, H. F. & CUTLER, S. J. (1955) Morbidity from Cancer in the United States. Part I. Variation in Incidence by Age, Sex, Race, Marital Status and Geographic Region. *Publ. Hlth Monogr.*, **29**, 1.

HUTT, M. S. R. & BURKITT, D. (1965) Geographical Distribution of Cancer in East Africa: a New Clinicopathological Approach. *Br. med. J.*, ii, 719.

JOJOT, C. & LAIGRET, J. (1922) Un cas de Tumeurs Superficielles Multiples Observe au Cameroun. *Bull. Soc. Path. exot.*, **15**, 956.

KAPOSI, M. (1872) Idiopatisches Multiples Pigmentsarkom der Haut. *Archs Derm. Syph.*, **4**, 265.

MACLEAN, C. M. (1963) Kaposi's Sarcoma in Nigeria. *Br. J. Cancer*, **17**, 195.

MCCRAE, A. W. R., PIKE, M. C. & SEMAKULA, E. (1968) In *Cancer in Africa*. Ed. P. Clifford, C. A. Linsell and G. L. Timms. Nairobi: East African Publishing House.

MOERTEL, C. G. (1966) Multiple Primary Malignant Neoplasms. In *Recent Results in Canccr Research*. Berlin: Springer-Verlag.

MURDOCK, G. P. (1959) *Africa, Its People and their Culture History*. New York: McGraw Hill Co.

MUSSINI-MONTPELIER, J. (1953) Angioréticulo-endothélio-sarcomatose de Kaposi en Afrique du Nord. *Acta U. int. contra. Cancrum*, **9**, 353.

OETTLÉ, A. G. (1962) Geographical and Racial Differences in the Frequency of Kaposi's Sarcoma as Evidence of Environmental or Genetic Causes. *Acta U. int. contra. Cancrum*, **18**, 330.

ROGOFF, M. G. (1968) Kaposi's Sarcoma. Age, Sex and Tribal Incidence in Kenya. In *Cancer in Africa*. Ed. P. Clifford *et al*. Nairobi: East African Publishing House.

SLAVIN, G., CAMERON, H. M., FORBES, C. & MORTON-MITCHELL, R. (1970) Kaposi's Sarcoma in East African Children: a Report of 51 Cases. *J. Path.*, **100**, 187.

SLAVIN, G., CAMERON, H. M. & SINGH, H. (1969) Kaposi's sarcoma in Mainland Tanzania: a Report of 117 cases. *Br. J. Cancer*, **23**, 349.

SUTHERLAND, T. W. (1958) In *Cancer*, Vol. 2. Ed. R. W. Raven. London: Butterworth.

TAYLOR, J. F., TEMPLETON, A. C., KYALWAZI, S. K. & LUBEGA, A. (1971) Kaposi's Sarcoma in Pregnancy. Two Case Reports. *Br. J. Surg.*, **58**, 577.

TAYLOR, J. F., TEMPLETON, A. C., VOGEL, C. L., ZIEGLER, J. L. & KYALWAZI, S. K. (1971) Kaposi's Sarcoma in Uganda: a Clinico-pathological Study. *Int. J. Cancer*, **8**, 122.

TEMPELTON, A. C. & VIEGAS, O. A. C. (1970) Racial Variations in Tumour Incidence in Uganda. *Trop. geogr. Med.*, **22**, 431.

THIJS, A. (1957) L'angiosarcomatose de Kaposi au Congo belge et au Ruanda-Urundi. *Annls Soc. belge Méd. trop.*, **37**, 295.

UGANDA GOVERNMENT (1967) *Atlas of Uganda*. Uganda: Department of Lands and Surveys.

VIANNA, N. J., GREENWALD, P. & DAVIES, J. N. P. (1971) Extended Epidemic of Hodgkin's Disease in High-school Students. *Lancet*, i, 1209.

WILLIAMS, E. H. & WILLIAMS, P. H. (1966) A Note on the Apparent Similarity in the Distribution of Onchocerciasis, Femoral Hernia and Kaposi's Sarcoma in West Nile. *E. Afr. med. J.*, **43**, 6.